# Calmodulin Interaction Interface with Plasma Membrane Ca^2+^-ATPase Isoforms: An Integrative Bioinformatic Analysis

**DOI:** 10.3390/ijms262311750

**Published:** 2025-12-04

**Authors:** Miguel Martínez-Fresneda, Esteban Lizano, Gabriela Echeverría-Garcés, Andres Herrera-Yela, Danna Feijóo, Grecia Victoria Vivas-Colmenares, Alvaro López-Zaplana, Leda Pedelini, Marta Mendoza, Juan Carlos Navarro, Jose Ruben Ramírez-Iglesias

**Affiliations:** 1 Emerging and Neglected Diseases Group, Health Sciences Faculty, Universidad Internacional SEK (UISEK), Quito 170120, Ecuador; mestre.martinez@uisek.edu.ec (M.M.-F.); esteban.lizano@uisek.edu.ec (E.L.); manuel.herrera@uisek.edu.ec (A.H.-Y.); danna.feijoo@uisek.edu.ec (D.F.); juancarlos.navarro@uisek.edu.ec (J.C.N.); 2 Faculty of Architecture and Engineering, Universidad Internacional SEK, Quito 170102, Ecuador; 3 Experimental and Applied Biomedicine Research Group, Health Sciences Faculty, Universidad Internacional SEK (UISEK), Quito 170120, Ecuador; maria.echeverria@uisek.edu.ec; 4 Latin American Network for the Implementation and Validation of Clinical Pharmacogenomics Guidelines (RELIVAF-CYTED), Santiago 8350499, Chile; 5 Grupo EXPRELA, CICA-Centro Interdisciplinar de Química y Biología, Departamento de Biología, Faculta de Ciencias, Universidad de la Coruña, Campus de Elviña, 15071 A Coruña, Spain; 6 School of Medicine, Universidad Internacional SEK, Quito 170102, Ecuador; grecia.vivas@uisek.edu.ec; 7 R&D Department, 3A Biotech, 30565 Murcia, Spain; alvaro.lopez9@um.es; 8 Máster en Epidemiología y Salud Pública, Universidad Internacional de Valencia (VIU), 46002 Valencia, Spain; lpedelini@universidadviu.com; 9 Centro de Estudios Biomédicos y Veterinarios (CEBIV), Instituto de Estudios Científicos y Tecnológicos (IDECYT), Universidad Nacional Experimental Simón Rodríguez (UNESR), Caracas 47925, Venezuela; mendozamarta17@gmail.com

**Keywords:** plasma membrane calcium ATPases, calmodulin binding domain, calcium disorders, calcium homeostasis

## Abstract

Plasma membrane Ca^2+^-ATPases (PMCA) are activated by calmodulin (CaM) via a C-terminal calmodulin-binding domain, CaMBD. Although specific mutations in this domain have been linked to disease, the broader impact of alternative substitutions across the interface remains unexplored. We applied an integrative in silico workflow to test six substitutions within CaMBD positions 1–18, L5R, N6I, I8T, V14E/D, and F18S, across PMCA isoforms 1–4. CaMBD sequences were aligned across isoforms, and candidates for substitutions were selected by conservation and nucleotide feasibility, prioritizing conserved or co-evolutionarily relevant sites, with substitutions possible by single-nucleotide change. PolyPhen-2 screened the impact of the substitutions on the protein functionality, the DisGeNET database was used to contextualize *ATP2B* genes with clinical phenotypes, and structural models plus binding free energy changes were estimated with AlphaFold3, FoldX, and MutaBind2. Effects were isoform and subregion dependent, with the strongest weakening toward the CaMBD C-terminus. V14E/D and F18S showed the largest and consistent predicted destabilization, consistent with disruption of conserved hydrophobic anchors. I8T and L5R had mixed outcomes depending on isoform, while N6I presented various scenarios with no clear effect. PolyPhen-2 classified most tested substitutions as damaging. Gene-disease evidence linked *ATP2B* to neurological, endocrine, and oncologic phenotypes, consistent with roles in Ca^2+^ homeostasis. Overall, CaMBD appears highly sensitive to perturbation, with distal positions 14–18 particularly vulnerable to substitutions that can destabilize CaM binding and potentially impair PMCA-mediated Ca^2+^ clearance in susceptible tissues.

## 1. Introduction

Ionized calcium (Ca^2+^) functions as a versatile second messenger, translating diverse extracellular signals into transient elevations of cytosolic Ca^2+^. These signals coordinate various processes, including fertilization, apoptosis, muscle contraction, cell proliferation, protein secretion, and neurite growth, among others [1]. Cells maintain a Ca^2+^ gradient to enable this signaling: resting cytosolic Ca^2+^ is kept near ~100 nM, roughly 2000 to 20,000-fold lower than the extracellular concentration, through the coordinated action of Ca^2+^ binding proteins, ion channels, and pumps distributed across intracellular organelles and the plasma membrane [2,3].

Among the mechanisms that preserve intracellular Ca^2+^ homeostasis, the plasma-membrane Ca^2+^-ATPase (PMCA) is the high-affinity pump that uses ATP hydrolysis to extrude a single Ca^2+^ ion in exchange for two H^+^ ions. PMCA belongs to the P2B-ATPase subclassification due to its autophosphorylation mechanisms during the catalytic cycle and the presence of an auto-regulatory region located at the C-terminal domain of the protein in animals and other eukaryotic organisms [4,5]. The pump activity contributes to returning cytosolic Ca^2+^ to its basal concentration and shapes Ca^2+^ signaling within the subplasmalemmal microdomains [6,7]. Multiple PMCA isoforms are expressed at distinct levels across human tissues, with the presence of over 30 molecular variants generated through alternative splicing. PMCA1 and PMCA4 are considered housekeeping pumps because they are present in nearly every tissue. However, their functions are not interchangeable. Deletion of PMCA1 is embryonically lethal, whereas PMCA4-null mice survive but lack the hyperactivated Ca^2+^ bursts required for specific cellular processes, highlighting the non-redundant roles of these proteins [8,9]. In contrast, PMCA2 and PMCA3 are highly enriched in excitable tissues of the central nervous system, including presynaptic terminals, cochlear outer hair cells, brain and spinal cord, where their fast kinetics and high basal activity fine-tune Ca^2+^ dynamics within specialized neuronal systems [8,9].

PMCA isoforms present differences across the protein sequences, one of them related to the specific splice site denominated “C” that is located at the Calmodulin Binding Domain (CaMBD) of the pump, at the C-terminal domain of the protein, which confers differential regulatory properties to the spliced isoform variants [10]. Calmodulin (CaM) is a ubiquitous Ca^2+^ sensor that plays a central role in Ca^2+^-dependent signaling pathways. The binding of Ca^2+^ to its four EF-hand sites, two in the N-terminal lobe and two in the C-terminal lobe, induces a transition from a compact (apo-CaM) to an open conformation (holo-CaM). The CaM-Ca^2+^-bound state exposes hydrophobic surfaces enriched in methionine residues that interact with amphipathic helices in a broad spectrum of target proteins, thereby coupling transient Ca^2+^ intracellular concentrations to downstream cellular responses [11,12,13]. The CaM interacts with its targets via the Ca^2+^-dependent CaMBD, which are usually composed of an alpha helix region with amphipathic properties and hydrophobic amino acid residues spaced in the form of motifs of different lengths, 1–10, 1–14, up to 1–18 [14,15,16,17].

CaM interacts with PMCAs via a 1–18 motif in the C-terminal domains, which induces a stimulation of the enzymatic activity of the pump through the liberation of the autoinhibitory state, in response to an increase in intracellular Ca^2+^ concentration [17,18]. This interaction could be affected by the length of the peptide related to the CaMBD and the substitution of specific hydrophobic anchor residues, which hampers the binding between CaM and PMCA-CaMBD. By following this strategy, the specific binding region of the pump has been characterized in humans [19,20], plants [21,22], and even unicellular parasites [23]. More recently, the physiological relevance of a stable binding between CaM and its interaction domain in PMCA has been highlighted in the descriptions of diseases related to point mutations in the CaMBD. Pathogenic variations in the CaMBD of PMCA, specifically G1107D in PMCA3 and V1143F in PMCA2, have been associated with cerebellar ataxia [24]. The PMCA3 G1107D mutation slows the decay of agonist-evoked cytosolic Ca^2+^ transients, indicating reduced Ca^2+^ extrusion, which alters the CaM-dependent regulation of the pump and impairs Ca^2^ recovery to baseline [25]. In PMCA2, the V1143F mutation within the CaMBD weakens the interaction between both proteins and compromises Ca^2+^ extrusion, also identified in a patient with congenital cerebellar ataxia [26].

Despite extensive functional characterization of CaMBDs across PMCA isoforms, the impact of point substitutions in the CaM-PMCA interface remains undefined, leaving open the question of how additional residues modulate pump activation. In this context, bioinformatic resources enable multi-scale analyses of molecular interactions and their potential relevance for diseases. These bioinformatic approaches are consistent with contemporary clinical variant classification practice, in which standardized frameworks such as the joint American College of Medical Genetics and Genomics-Association for Molecular Pathology (ACMG-AMP) guidelines integrate multiple lines of evidence, including bioinformatic data, into evidence-based pathogenicity categories [27]. Within this framework, in silico predictions are formalized as computational evidence codes such as PP3, which reflects multiple lines of computational evidence supporting a deleterious effect on the gene or gene product and thereby contributes to the overall assessment of potential clinical impact. Accordingly, the primary aim of this study was to employ an integrative bioinformatics approach to assess the influence of six rationally selected substitutions on interactions between the CaMBD of different PMCA isoforms and CaM, and to relate these effects to potential diseases.

## 2. Results

### 2.1. Identification of PMCA Isoforms/Variants, CaMBDs, and Definition of Amino Acid Substitutions to Evaluate

Sequences for each of the four human PMCA isoforms (PMCA1-PMCA4) were retrieved from the GenBank database. In addition to the accession numbers corresponding to the main isoforms, alternative splice variants were also identified, resulting in more than 30 sequences for PMCAs 1–4. In many cases, CaMBD was identical among different variants of the same isoform. Therefore, these redundant sequences were excluded, and only the non-redundant variants for each isoform are listed, along with the Accession codes, as presented in the Supplementary Materials File S1. These sequences are retained for comparative alignment analyses and subsequent structural modeling.

**Figure 1:**
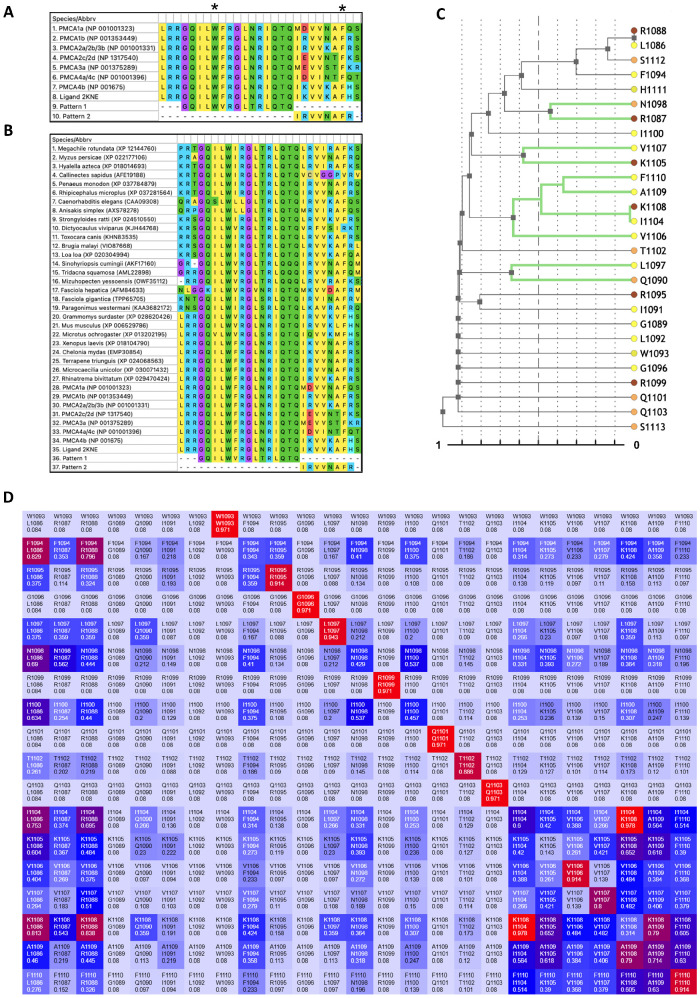
Analysis of amino acid positions within the Plasma membrane Ca^2+^-ATPase (PMCA) calmodulin-binding domains (CaMBD). (**A**) CaMBD delimitation of human PMCA isoforms/variants. There is only one accession number presented in case of multiple isoforms with the same CaMBD. The delimited motif within the CaMBD is indicated by the main anchors of the aromatic residues W1 and F18 (black asterisks). (**B**) PMCA CaMBD multiple sequence alignment (MSA) of multiple sequences of different organisms. (**C**) CoeViz dendrogram of alignment rows clustered by covariation. Rows or branches highlighted in green denote stronger covariation pairs of residues, within the 1–18 motif. Colored circles at the tips encode residue chemistry from the CoeViz/POLYVIEW-2D web server. (**D**) CoeViz heat map of phylogeny-weighted mutual information. Rows and columns correspond to positions within the 1–18 CaMBD segment (numbered according to 2KNE/PMCA4b). Each off-diagonal cell reports the covariation score for that pair of positions: Warmer (towards red) and cooler (towards blue) colors indicate higher and lower covariation, respectively, related to the scores of the phylogeny-weighted mutual information and displayed as normalized values (0–1). Cell labels indicate the residue/position identifiers for the row and column. The main diagonal reflects per-position variability and is not interpreted as covariation.

To characterize conservation within human PMCA CaMBDs and identify covarying residue clusters across orthologs, we compiled CaMBD sequences from human PMCAs and diverse metazoan species, generated a multiple sequence alignment (MSA), and performed a phylogeny-weighted mutual-information co-evolution analysis (Figure 1). The human CaMBD alignment revealed highly conserved amino acids at the N-terminus of the domain of the sequences, which also present good alignment with both the 2KNE ligand (LRRGQILW_1093_FRGLNRIQTQIKVVKAF_1110_HSS) [18] and the CaM-recognition Pattern 1 (GQILWFRGLNRIQTQ). The sequence located at the C-terminus presents more variability between PMCAs and Pattern 2 (IRVVNAFR) [28] (Figure 1A). The MSA with the ortholog sequences presented similar results, with a slight increase in variations in both segments of the Patterns 1 and 2 (Figure 1B). The CoeViz dendrogram, which hierarchically clusters alignment rows by covariation, resolves two regions within the 1–18 CaMBD segment, spanning W1 (1093) to F18 (1110). In Pattern 1, at the N-terminal half, L5 (1097) and N6 (1098) emerge among the most distinctive leaves, showing covariation with positions just outside the 1–18 window, such as Q1090 and R1087. In Pattern 2, at the C-terminal half, a compact cluster comprises V14 (1106), I12 (1104), K16 (1108), A17 (1109), and F18 (1110), with an additional small node linking V15 (1107) and K13 (1105) (Figure 1C). The phylogeny-weighted mutual-information heat map reveals signals of high covariation across Pattern 2. Within this area, V14 and F18 participate in multiple highlighted pairs, consistent with maintenance of the hydrophobic interface recognized by CaM. By contrast, Pattern 1 shows a weaker and more localized signal, consistent with higher conservation relative to Pattern 2 (Figure 1D). In summary, Figure 1 indicates that within Pattern 2, or the second half in the CaMBDs, there are several highly covariant positions, exhibiting a concentrated covariation signal, suggesting these sites as particularly consequential for the recognition of CaM.

To prioritize substitutions for structural analysis, we examined codons encoding conserved positions in human CaMBDs together with residues highlighted by the co-evolution analysis. The positions that met all criteria were L5, N6, I8, V14, and F18. For the codons present, each site can be altered by a single nucleotide change at the second codon position (Table 1). For example, N6: Asn → Ile (AAT/AAC → ATT/ATC), I8: Ile → Thr (ATC/ATT → ACC/ACT), F18: Phe → Ser (TTC/TTT → TCT/TCC). As shown in Table 1, the V14 position yielded different results dependent on the isoform studied, thus resulting in acid aspartic and acid glutamic substitutions. Integrating the alignment and co-evolution results with this nucleotide-feasibility analysis, we selected six substitutions within the 1–18 Ca^2+^-dependent CaMBD motif: three in Pattern 1, L5R, N6I, I8T, and three in Pattern 2, V14E/D, and F18S.

**Figure 2:**
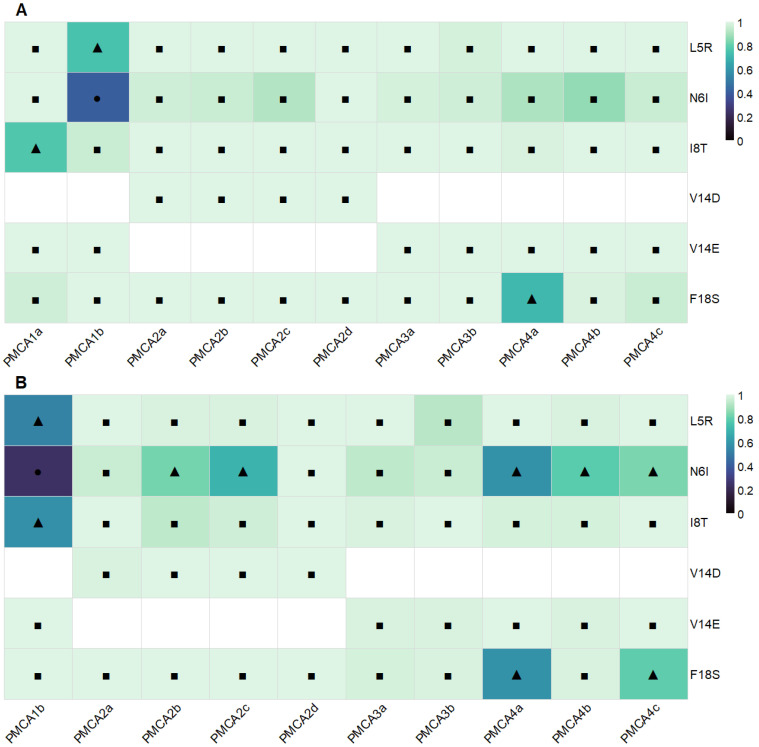
Potential impact of substitutions evaluated on the PMCAs functionality. Heatmaps show PolyPhen-2 predictions for CaMBD substitutions across PMCA isoforms. Color encodes the PolyPhen-2 score (0–1). Symbols indicate categories related to the PolyPhen-2 scores (0–1): ● benign (<0.2), ▲ possibly damaging (0.2–0.85), ■ probably damaging (≥0.85), numerical ranges derived from the results of the server. Results for HumDiv and HumVar models are shown in panels (**A**) and (**B**), respectively. Substitutions (axis Y) are ordered by position within the 1–18 CaMBD motif, and the PMCA isoforms are presented on the X axis. White spaces: substitutions not evaluated in the specific isoforms.

PolyPhen-2 analysis was performed on the full-length protein sequences of all PMCA isoforms shown in Figure 1 to obtain a preliminary estimate of the functional impact of the proposed CaMBD substitutions (Figure 2). Across PMCA1–4, PolyPhen-2 classified nearly all substitutions as “probably damaging”, with a smaller subset as “possibly damaging”, and a single one classified as “benign” in PMCA1b/N6I. Predictions were consistently concordant between the two models, HumDiv optimized for rare deleterious alleles and HumVar optimized for diagnostic settings (Figure 2A,B). Overall, these results indicate a high probability of functional negative impact of the substitutions proposed at the CaM-CaMBD interface. The complete dataset, including each variant score, model-specific categories, and the corresponding sensitivity and specificity values, is provided in the Supplementary Materials File S2.

### 2.2. Structural Analysis of PMCA CaMBD-CaM Complexes: Wild Type, Literature-Reported Mutations, and Tested Substitutions

Five structural models (Model 0–4) were generated using AlphaFold v3 for each one of the PMCA isoform/variants, including the wild-type (WT) forms, literature-reported mutations, and newly proposed substitutions, all modeled in complex with CaM. To assess prediction precision, 95% confidence intervals (95% CI) for the mean of the inter-residue predicted TM-score (ipTM) were calculated using Student’s t-distribution (Table 2).

Comparison between ipTM values showed that Model 0 consistently achieved the highest or near-highest scores across most complexes, indicating superior predicted structural reliability. The mean ipTM values for WT isoforms ranged from 0.52 to 0.74, with narrow 95% CI widths, generally below 0.1, confirming the reproducibility and internal consistency of AlphaFold v3 predictions. PMCA1b and PMCA4b displayed the highest mean scores, whereas PMCA2c/2d and PMCA3a showed lower and more dispersed confidence intervals, suggesting greater conformational flexibility at their CaM-binding interfaces.

Models for literature-reported mutations maintained comparable reliability, with mean ipTM 0.63–0.71, indicating that the experimentally observed substitutions did not markedly destabilize the CaM-PMCA interaction geometry.

In contrast, the proposed substitutions exhibited greater variability (ipTM 0.33–0.81), reflecting diverse effects on structural confidence. Variants such as I8T and L5R retained high ipTM values, implying preserved binding compatibility. In contrast, V14E/D substitutions produced the lowest scores and widest IC_95_ ranges, consistent with local destabilization and increased flexibility within the CaMBD interface.

Regarding the structural Root Mean Squared Deviation (RMSD) analysis, values were computed only for PMCA4b, as it is the only isoform with an experimentally resolved CaM-CaMBD complex, using the PDB 2KNE Nuclear Magnetic Resonance (NMR) as the reference structure. RMSDs were ~2.0 Å for Models 0–4 (2.458 for Model 0 and 2.658 for Model 4). These values support good global agreement with the experimental conformation and suggest that the predicted complexes are structurally possible, especially at the interaction core.

**Figure 3:**
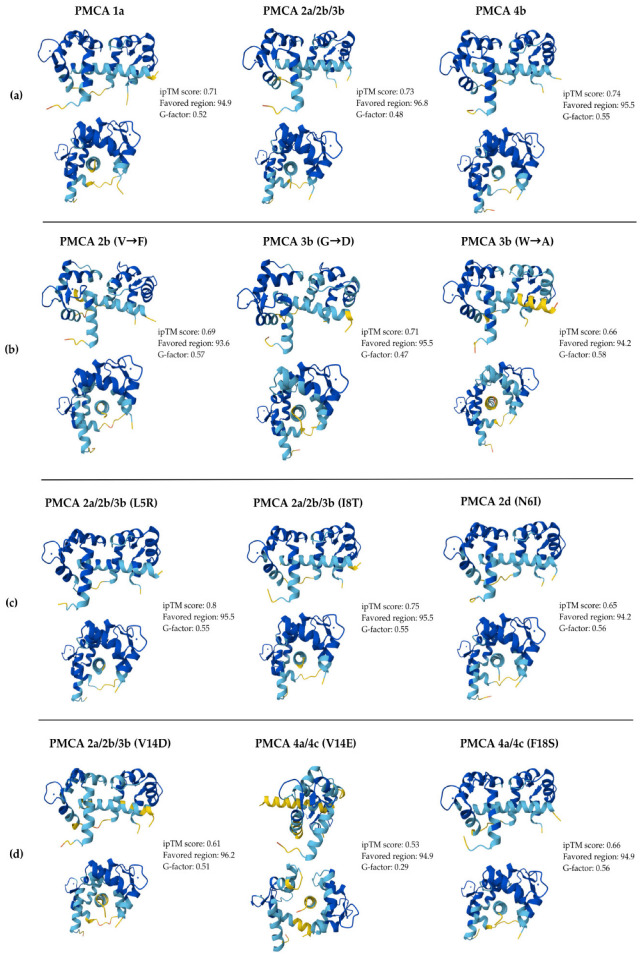
Representative models of the CaM-CaMBD complexes predicted using AlphaFold v3. All structures are presented with a lateral and frontal view. For each model, quality metrics are reported, including ipTM, Ramachandran percentages of residues in favored regions, and G-factor. Panel (**a**) displays the wild-type (WT) isoforms, which serve as structural references. Panel (**b**) includes the mutations previously reported in the literature. Panels (**c**,**d**) present the representative models of the substitutions proposed in this study. The colors indicate the local per-residue measure confidence (pLDDT) level of the predicted models according to AlphaFold v3: dark blue (very high, pLDDT > 90), light blue (high, 90 > pLDDT > 70), yellow (low, 70 > pLDDT > 50), and orange (very low, pLDDT < 50).

Figure 3 displays representative poses for all CaM-CaMBD complexes evaluated. The WT models, shown in front and side views, adopt the canonical conformation in which holo-CaM wraps around the amphipathic CaMBD helix. Isoform-specific models present slight differences in both the receptor and the ligand (Figure 3a). For the literature-reported mutation models, the most notable deviations compared to WT are small displacements and rotations of the CaMBD helix within the CaM binding space, best appreciated in the side views (Figure 3b). The complexes with the proposed substitutions generally preserve the WT conformation (Figure 3c,d), with the clearest difference observed for the V14E/D substitution in PMCA4c and PMCA2a_2b_3b, which alters peptide orientation and local packing at the interface (Figure 3d). In general, the structures are maintained and comparable with WT models, except for the mentioned specific models, indicating that the overall conformation is maintained despite alteration in the alpha helix derived from the substitutions. Consistently, high Ramachandran favored region percentages indicate that most backbone φ/ψ angles fall in energetically preferred conformations, while the positive G-factors reflect overall stereochemical normality. These values support the structural feasibility of the displayed models. Complete sets of predicted conformations, poses, and Ramachandran plots for all complexes are provided in the Supplementary Materials Files S3 and S4.

### 2.3. Energetic Characterization of CaM-CaMBD Complexes

ΔG values of the CaM-CaMBD complexes were calculated using FoldX for both WT and reported mutant PMCA isoforms. For WT complexes, ΔG values ranged from −48.22 to −61.92 kcal/mol. The most favorable energy was observed for PMCA2a_2b_3b (−61.92 kcal/mol), followed by PMCA4b (−59.20 kcal/mol), while PMCA1a exhibited the highest value of −48.22 kcal/mol (Supplementary Materials File S5).

For the complexes with reported mutations, ΔG values were −59.94 kcal/mol for PMCA2b V → F, −46.04 kcal/mol for PMCA3b W → A, and −50.65 kcal/mol for PMCA3b G → D (Supplementary Materials File S5), which correspond to a variation of binding energy, calculated with FoldX, of 1.98, 15.88, and 9.29, respectively (Table 3). Despite the differences of magnitude, the MutaBind2 ΔΔG for these complexes were consistent with the FoldX values based on positive final variation of the Gibbs energy function (Table 3). These results indicate that the applied methods consistently predict an increase in Gibbs free energy across all naturally mutated complexes, suggesting a potential destabilization of the CaM-CaMBD interaction.

Regarding the FoldX predicted binding energy changes for the modeled CaM-CaMBD complexes, most substitution combinations yielded positive ΔΔG values and exceeded the ±1.7 kcal/mol cutoff, consistent with predicted binding weakening (Table 3). Within Pattern 1, ΔΔG values were mixed, showing cases of both predicted weakening and strengthening of the CaM-CaMBD interaction. In contrast, Pattern 2 showed a clearer overall tendency toward binding weakening. Notably, V14E/D consistently produced large positive ΔΔG values across interfaces, with the highest increases in the PMCA4 complexes (12.05–16.12 kcal/mol), indicative of loss of affinity. For MutaBind2, all ΔΔG estimates were positive, and thus none of the evaluated substitutions were predicted to strengthen binding. Consistent with FoldX, the largest ΔΔG shifts were observed for Pattern 2 substitutions, which exceeded the ±1.5 kcal/mol MutaBind2 cutoff (Table 3). By position, N6I exhibited the strongest method dependence: FoldX predicted stabilization in some isoforms, whereas MutaBind2 returned neutral to mildly destabilizing values, indicating that the direction and magnitude of its effect are less robust across estimators. Overall, the tested substitutions disrupted binding in PMCA4b and in the PMCA2a/2b/3b complex in most cases (Table 3). Across both ΔΔG approaches and all isoform substitution combinations (n = 70), only fourteen estimates fell within the respective method-specific cutoffs, supporting that this interface region is highly sensitive to the amino acid substitutions evaluated.

### 2.4. Potential Impact of Ca^2+^ Disruption Related to PMCA Genes in Pathological Phenotypes

**Figure 4:**
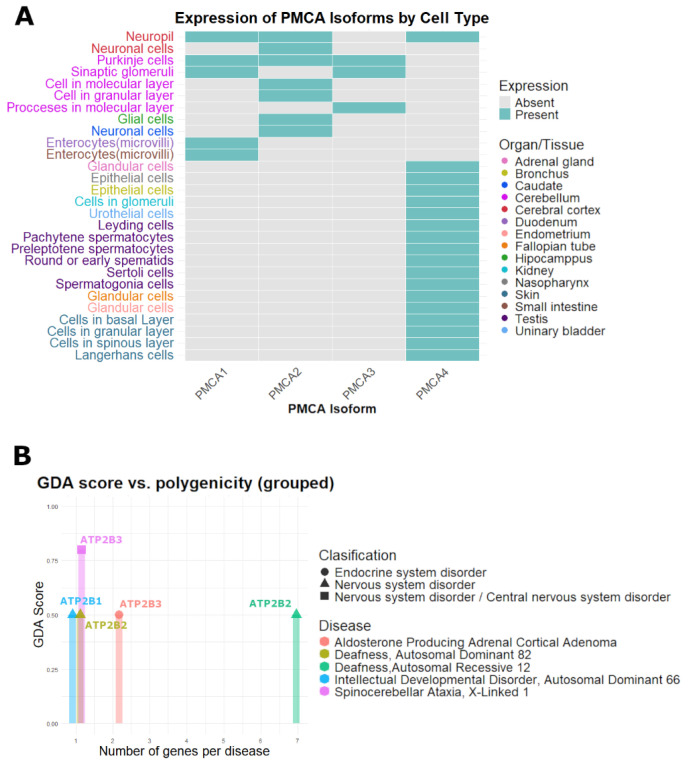
Distribution of PMCA isoforms and diseases related to the *ATP2B* genes. (**A**) PMCA isoforms with high levels of protein expression, located in cells and tissues of the human body. (**B**) Classification of disease with correlation with the *ATP2B* genes.

Figure 4A displays a presence/absence heatmap of tissues and cell types with a high protein expression for the four PMCA isoforms. PMCA1-PMCA3 show a more restricted pattern, concentrated in neuronal populations from the cerebellum and cerebral cortex, whereas PMCA4 exhibits a broader distribution that spans various systems, including nervous, endocrine, respiratory, digestive, and reproductive tissues (Figure 4A). Figure 4B is a summary of the five diseases associated with PMCA coding genes *ATP2B1*-*ATP2B3* after filtering by gene-disease association (ScoreGDA) and under the “Causal Mutation” classification. Most gene-disease associations, 4 of 5, are at GDA = 0.5, with a single stronger association at 0.8 GDA for *ATP2B3*. Polygenicity is characterized by three monogenic diseases, one oligogenic disease involving two genes, and one additional polygenic disease involving seven genes. Disorders of the nervous system predominate, covering diseases such as spinocerebellar ataxia, deafness, and intellectual developmental disorder, alongside one endocrine condition, aldosterone-producing adrenal cortical adenoma (Figure 4B). There were no diseases related to *ATP2B4* that met the inclusion criteria. In the Supplementary Materials File S6, we enumerate all gene-disease associations retrieved for *ATP2B1*-*ATP2B4*, including entries classified as “GeneticVariation” and with high polygenicity of total number of genes linked to that disease (NgenesD) > 30. Some results do not pass the filters, but may indicate emerging roles of *ATP2B* genes, such as the malignant neoplasm of prostate associated with *ATP2B4*, muscle hypotonia associated with *ATP2B3*, and epileptic encephalopathy associated with *ATP2B2* (Supplementary Materials File S6).

## 3. Discussion

In this study, we assessed the in silico impact of six CaMBD substitutions across human PMCA isoforms as well as their potential effect on altering pump function and pathological phenotypes. Part of our bioinformatic workflow relied on AlphaFold3 to generate models for the CaM-CaMBD complexes, followed by structural confidence and energetic assessments. For WT and literature-reported mutations, the predicted complexes exhibited conformations with canonical poses across five predictions per complex, with 95% CI ipTM values typically falling within the 0.70–0.60 range. The lowest confidence was presented in the predictions of the evaluated substitutions located at the C-terminal or within the Pattern 2 region of the CaMBDs. AlphaFold3 is a diffusion-based model trained on PDB structures that predicts atomic coordinates for several types of complexes and often outperforms classical docking [29]. Nonetheless, AlphaFold3 confidence metrics do not quantify binding affinity, and mutation-induced rearrangements or local flexibility can lower pLDDT without establishing a definitive structural change. Therefore, we complemented these predictions with PolyPhen-2 and independent evidence from the literature. FoldX and MutaBind2 binding energy analyses indicated that all tested models for the literature-reported mutations produced positive ΔΔG values, consistent with less favorable and less stable CaM-CaMBD complexes. This trend aligns with experimental reports for PMCA3 G1107D and PMCA2 V1143F, as well as the PMCA3 W1104A control, which weakens CaM binding and increases the dissociation constant, ultimately affecting Ca^2+^ extrusion and elevating the intracellular cytosolic Ca^2+^ concentration [6,25,26]. In contrast, conventional molecular docking with the HawkDock server, coupled with PEPFOLD-4 for ligand modeling, did not reproduce this trend. ΔΔG estimates for literature-reported mutations were inconsistent, showing both positive and negative changes in binding energy (Supplementary Materials File S7), which was the basis to fund the complex modeling only with AlphaFold within our bioinformatic workflow. Additionally, AlphaFold3 has been used to evaluate the impact of missense substitutions or mutations across diverse biomedical contexts, including the *EFNB1* gene in craniofrontonasal syndrome [30], Phosphomannomutase-2 in congenital disorders of glycosylation [31], and the *NF1* gene in neurofibromatosis type 1 [32]. In these studies, similar to our approach, AlphaFold3 models were treated as an in silico hypothesis generation and were benchmarked using various quality metrics, such as ipTM, RMSD, together with predefined thresholds for ΔΔG and classifying scores.

Concerning the ΔΔG results for models of the evaluated substitutions, most models showed a weakening of CaM binding to the PMCA CaMBD. A clear difference emerged between changes in Pattern 1, the segment closer to the N-terminus, and Pattern 2, the segment closer to the C-terminus. These patterns, used in our study as an additional sequence for CaMBD delimitation, correspond to a segmentation within the CaMBD derived from evolutionary analysis that shows higher amino acid conservation toward the N-terminus and greater sequence variability toward the C-terminus in PMCAs from humans and other organisms [28]. Under this context, the mixed responses, mainly weakening with occasional binding strengthening, could be attributed to isoform-specific sequence differences concentrated in Pattern 2. Related to this matter, CaM presents a remarkable binding plasticity, repositioning its N- and C-lobes and flexible linker to engage α-helices in diverse topologies [33]. This versatility is exemplified in nuclear resonance magnetic data for PMCA4b, with its hydrophobic pockets covering the CaMBD anchors [18], in calcineurin, adopting an elongated head-to-tail dimer encircling a single target peptide [34], and in melittin complexes that lack classical anchoring interactions, yet maintaining nanomolar affinity [35]. With this background, isoform-specific sequence differences, especially in distal CaMBD segments, may reduce the permissiveness for optimal CaM accommodation after missense substitutions, yielding a variety of effects.

The substitutions used to evaluate their effects on the molecular interface between CaM and the PMCA CaMBD exhibit different properties according to classical classification systems based on charge, polarity, and side-chain volume [36]. L5R and V14E/D are considered radical substitutions because they alter all three criteria. By contrast, N6I and I8T are conservative with respect to charge but radical with respect to polarity and volume, whereas F18S is radical due to the loss of hydrophobicity and a marked reduction in side-chain volume.

Among the positions tested, the phenylalanine residue stands out as one of the most critical. Substituting Phe with Val and truncating peptides that remove this residue have demonstrated that it serves as a principal anchor for CaM, essential for the affinity and stabilization of the CaM-CaMBD complex [20]. Although F18S is a different substitution, results across PMCA CaMBD isoforms indicate a destabilization of the complex that cannot be compensated by an alternative, energetically favorable rearrangement. This outcome is similar to the effect of an F → S substitution used to delineate the PMCA CaMBD in the unicellular parasite *Trypanosoma equiperdum* [23].

However, beyond the confirmed importance of phenylalanine, the other positions that form the configuration of the amino acids in the second half of the CaMBD are also functionally relevant for achieving full interaction with CaM. This is supported by studies using the C20 peptide (LRRGQILWFRGLNRIQTQIK), which does not reproduce the full extent of pump activation by CaM observed with the C28 sequence (LRRGQILWFRGLNRIQTQIKVVKAFHSS) [19,20,37]. Within this region, Pattern 2 or the second half of the CaMBD, V14 is present. In the FoldX and MutaBind2 variations energy values, V14E/D substitutions exhibited one of the largest increases in ΔΔG together with F18S, but also one of the lowest levels of confidence in AlphaFold models. Given that other substitution sets yielded higher ipTM values, we hypothesize that V14E/D represent highly detrimental substitutions that push the CaM-CaMBD interface outside the evolutionary and physicochemical compatibility captured by the model, thereby lowering ipTM. This may also be partly due to the closeness of other charged residues, such as D, R, E, and K, depending on the studied isoform, located in the 13th position, which may exercise a major repulsion or attraction to the V14E/D. Together with F18, V14 is among the most conserved residues in this segment, suggesting a limited capacity to adopt a compensatory rearrangement that would yield a new energetically favorable conformation across isoforms. In the classical nomenclature, this residue participates in the 1-5-8-14 hydrophobic motif, classifying it as one of the hydrophobic anchors that help secure globular lobes of CaM around the target peptide [14,38].

Although most of the effects we report weaken the interaction of CaM with its domain in the PMCA, the opposite scenario, mutations that strengthen a protein–protein interaction, can also be detrimental because they may compromise the precise control of Ca^2+^ oscillations in response to stimuli. For this reason, we also highlighted a ±1.7 kcal/mol cutoff for FoldX in cases where predicted ΔΔG decreased. Besides the PMCA and Ca^2+^ context, an example of how a substitution that results in strengthening of binding may be negative is the gain-of-function mutations in platelet integrin αIIbβ3. In this case, the mutations can lock the receptor in a high-affinity fibrinogen-bound state in murine models. Continuous ligand occupancy disrupts platelet structure-function and leads to bleeding phenotypes, as shown for β3 C560R mutations and other related high-affinity variants [39].

In recent years, the evidence of association between the *ATP2B* genes with several diseases has been steadily increasing, thanks to the molecular-clinical studies perspective and to the increasing implementation of biomedical databases and application of next-generation sequencing for medical cases. These approaches have paved the way to describe medical problems from a molecular point of view and to consider emerging conditions related to specific genes. Our screening in the DisGeNET biomedical database showed results such as ataxia, a disease with a clear link to the CaMBD mutation in the PMCA3 isoforms (*ATP2B3* gene) reported in the literature [6,25,26], which highlights the importance of this region, even more considering the high expression of this isoform in the nervous system. Beyond *ATP2B3*-PMCA3 CaMBD-linked ataxia, converging evidence across *ATP2B* genes strengthens the medical relevance of amino acid changes at the PMCA. For instance, for the *ATP2B2* gene, it has been reported that missense and frameshift variants are associated with a spectrum of neurological phenotypes, such as ataxia, dystonia, intellectual disability, autism, seizures, with functional assays [40], which are in concordance with our reported findings. In this study, two variants, V1113G and T1086D, are located at the C-terminal domain of the pump, near the CaMBD, and are related specifically to neurodevelopmental disorders [40].

Outside neurology, *ATP2B3* has been linked to aldosterone-producing adrenocortical adenomas, which are benign tumors of the adrenal cortex that secrete aldosterone autonomously and typically present with hypertension, hypokalemia, and muscle weakness. In these tumors, Beuschlein et al., identified somatic in-frame deletions in the M4 transmembrane helix of PMCA3, alterations predicted to impair Ca^2+^ coordination at the transport site, a rise in cytosolic Ca^2+^, and thereby drive autonomous aldosterone production [41]. Besides the list of diseases retrieved under our DisGeNET inclusion criteria, additional pathologies deserve attention for their involvement in other phenotypes. In the case of PMCA4, this pump has emerged as a modifier of cancer from the cell migration and metastasis point of view, suppressing dissemination in melanoma and gastric models, while being upregulated in pancreatic ductal adenocarcinoma [42]. Given that CaM is one of the principal activators of PMCA and that CaM-PMCA binding kinetics are isoform and variant-specific, substitutions within the CaMBD that perturb CaM-dependent activation provide a possible route from molecular dysregulation to altered migratory behavior and metastatic potential. Although only a part of the clinically oriented research establishes a direct link between the CaMBD and pathological phenotypes, converging evidence across *ATP2B* genes indicates that perturbations to this regulatory domain can contribute to disease in an isoform and tissue-dependent manner, where even small disturbances may have physiological consequences. Finally, consistent with this view, PolyPhen-2 classified all substitutions proposed in this study as probably damaging or possibly damaging, indicating that these changes in the great majority of cases may result in intracellular Ca^2+^ imbalance and be followed by serious effects at systemic level.

In general, the isoform results underscore a context dependence, in which PMCA1–4 differ in tissue distribution and physiological roles, so the same CaMBD substitution need not yield the same molecular outcome across isoforms. This heterogeneity provides a rationale for case stratification by *ATP2B* gene or isoform and by the segment of the CaMBD affected, potentially predicting distinct phenotypes and follow-up needs. Within clinical variant interpretation practice related to the nomenclature and codes of the ACMG guidelines [27], all the convergent computational evidence here reported supports applying the PP3 code to variants that alter sensitive CaMBD positions, while the signal concentration in the C-terminal half or Pattern 2 justifies the PM1 code (variant in critical domain with low tolerance to change). Consistent with this guideline specification, it is possible that the detrimental effects of the substitutions in specific PMCA isoforms shown here can be classified as high-impact functional, in order to subsequently look for a classification of variant of uncertain significance (VUS) within the *ATP2B* genes. Future integration of evidence related to population rarity, validated functional assays, and alternate missense at the same residue could advance classification toward more informative categories [27,43]. Additionally, our approach aligns with recommendations from recent methodological work on variant impact predictors by combining complementary in silico scores with structural context and calibrating their interpretation to the specific biological question and scale, thereby strengthening the translational utility of these in silico findings [44].

The main limitation of this work is its intrinsic in silico design, which requires validation both in vitro and through clinically relevant descriptions in patients with the corresponding substitutions. A second limitation concerns a complementary side of CaM regulation, specifically the intramolecular receptor for the CaMBD [45,46]. We did not explore models for this interface; yet, changes in this region of the pump could mimic a constitutively activated state independently of CaM binding. Despite this context, the predictions offered here provide a focused analysis of how possible nucleotide variants leading to specific amino acid substitutions across the CaMBD architecture of multiple PMCA isoforms and cell types might perturb CaM-dependent activation. The magnitude of Ca^2+^ dysregulation caused by these CaMBD mutations, and how it compares with mutations in other regulatory or transport domains of the pump, remains to be determined experimentally.

## 4. Materials and Methods

### 4.1. Design of the In Silico Approach

**Figure 5:**
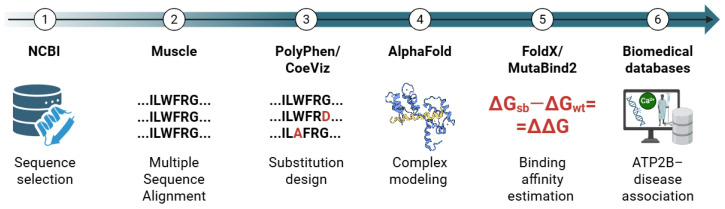
Schematic workflow of the steps and resources employed in this study. Figure created with Biorender.

A six-step, fully in silico pipeline (Figure 5) was implemented to (1) identify and retrieve human PMCA isoforms and splice variants, (2) align the protein sequences, delimit the CaMBD to register unique sequences across the different variants, and define native wild-type (WT) sequences, (3) perform a co-evolution analysis and define point substitutions in key residues in the CaMBD and evaluate potential impact on the protein functionality, (4) predict CaM-CaMBD complex structures, (5) estimate binding affinity changes (ΔΔG) in the evaluated system, and (6) integrate molecular findings with disease-oriented resources. All steps were documented to ensure reproducibility.

### 4.2. Retrieval of PMCA Isoform and Variant Sequences

A systematic query of NCBI GenBank (https://www.ncbi.nlm.nih.gov/gene (accessed on 20 June 2024)), in the NCBI Reference Sequences (RefSeq) section, was performed to retrieve protein sequences encoded by gene names related to the isoforms *ATP2B1*-*ATP2B4* (*Homo sapiens*). For each gene, the canonical PMCA isoform and annotated alternative C-terminal splice variants were identified. Accession numbers were verified and recorded. FASTA sequences were downloaded on 20 June 2024 and used as inputs for downstream analyses.

### 4.3. CaMBD Delimitation

To locate and register the CaMBDs in the PMCA isoform/variants, sequences were scanned exclusively for the C28 peptide of the PMCA4b, which is present in the NMR structure 2KNE [18]. The consensus evolutionary patterns, Pattern 1 (GQILWFRGLNRIQTQ) and Pattern 2 (IRVVNAFR), described by Mantilla et al. [28], were also used to delimit the region for analysis. The sequences were aligned with MUSCLE in MEGA X (Ver 12.1) using default parameters: Gap Open = −2.90, Gap Extend = 0, hydrophobicity multiplier = 120, maximum memory = 2048 MB, maximum iterations = 16, clustering method in phases 1 and 2 = UPGMA, minimum diagonal length (λ) = 24. For each isoform/variant, Wild-Type (WT) was defined as the unmodified sequence of the corresponding GenBank accession.

### 4.4. Definition and Rationale of Amino Acid Substitutions in the CaMBD of PMCA Isoforms

Amino acid substitutions were chosen within the core CaM-PMCA interaction motif, specifically between the 1–18 residues in the CaMBDs. Selection followed two complementary criteria. (1) Positional conservation and co-evolution clustering. We first prioritized residues strongly conserved across human PMCA CaMBDs, using a multiple sequence alignment (MSA). To increase the evolutionary signal, we then built an evolutionarily enriched MSA by adding 27 orthologous CaMBD sequences from multicellular eukaryotes (Mantilla et al., [28]). Co-evolution was assessed with the CoeViz web server (https://polyview.cchmc.org/ (accessed on 9 November 2025)) [47] to identify covarying clusters and nominate candidate residues whose variation is accompanied by putative compensatory substitutions elsewhere within the CaMBD. This analysis was anchored to the 2KNE PDB structure and analyzed the custom MSA with phylogeny-weighted mutual information (20-letter alphabet) to highlight rows with high covariation. (2) Nucleotide feasibility. For each prioritized position, we analyze the codons used by human PMCA1–4 sequences and selected substitutions that can be achieved by a single nucleotide change at the second position of the codon. When isoforms used different codons, feasibility was evaluated separately for each isoform. This choice is made based on the organization of the genetic code, where coding is driven primarily by the first two bases, whereas the third position is often degenerate and frequently yields synonymous changes [48], and the relevance of the first two bases during translation, highlighting the central role of the middle base in determining amino acid identity [49]. Therefore, editing the second position provides a reasonable route to realistic non-synonymous variants for structural testing. Additionally, each proposed PMCA substitution was then evaluated with PolyPhen-2 (http://genetics.bwh.harvard.edu/pph2/ (accessed on 15 April 2025)) [50,51] to obtain an initial prediction of the potential impact of the amino acid substitution on total protein structure-function. PolyPhen-2 combines multiple sequence and structure-based features in a Naïve Bayes classifier to estimate the probability that a missense substitution is functionally damaging, returning a numerical score between 0 (benign) and 1 (damaging) together with a qualitative category of benign, possibly damaging, and probably damaging, which is defined by preset false positive rate thresholds for each trained model (HumDiv and HumVar). In this study, we report predictions from both the HumDiv model, trained on Mendelian disease variants versus fixed differences in closely related mammals, and the HumVar model, trained on disease variants versus common polymorphisms. In both models, the “possibly” versus “probably” labels primarily reflect prediction confidence rather than graded effect size, based on the score established in the server. The “probably damaging” and “benign” predictions indicate high-confidence calls of deleterious and tolerated effects, respectively. The “possibly damaging” label denotes a lower-confidence prediction of a damaging effect [51].

### 4.5. Prediction of Molecular Structures and Protein-Peptide Interactions

Complexes between CaM and each CaMBD, including WT, literature-reported mutations, and substitutions evaluated, were predicted using the AlphaFold Server v3, which directly models biomolecular complexes rather than isolated proteins [29], reducing reliance on sequential homology modeling, docking, and refinement steps and thereby limiting potential error propagation. CaM was provided as the clean human amino acid sequence, specifying a Ca^2+^-bound state in the input, to simulate an holo-CaM state. For each CaMBD-CaM complex, five independent models were generated, and the top-ranked prediction was selected primarily by the interface predicted TM-score (ipTM). Also, pLDDT and pTM per model were saved. For each complex, we report the mean and 95% confidence intervals across the five models. As a first level of validation of the workflow implemented in this study, the 2kne PDB structure of the CaM-PMCA4b (C28) was used with the same models generated by the AlphaFold Server to calculate the root-mean-square deviation (RMSD) in the PyMOL visualization system (Ver 3.1).

### 4.6. Determination of Energy Interaction and Variation of the Free Energy of Gibbs

Generated complexes were processed with FoldX (Ver 5.0, foldx_20251231) [52,53] as follows: each structure underwent RepairPDB, followed by AnalyseComplex to obtain binding free energies in kcal/mol. For every WT/mutant or substitution pair, ΔΔG was computed as ΔΔG = ΔG_sbs − ΔG_wt, where ΔG_sbs corresponds to the ΔG of substitutions evaluated and ΔG_wt is the ΔG of Wild-Type structures. Positive resulting values indicate less favorable binding. As a second level of internal validation of the workflow, the interaction energies of the literature-reported isoform with mutations were also calculated and compared with their respective WT model. To highlight the effects related to the substitutions evaluated in the CaMBD-CaM complexes, we used a threshold or cutoff taken into account the FoldX error of 0.85 kcal/mol [53], multiplied by two. Therefore, a cutoff of ±1.7 kcal/mol was established for the ΔΔG calculated to consider the effect of the substitution proposed on the ligand-receptor evaluated. In addition, ΔΔG values were independently estimated with the MutaBind2 web server [54], which only requires the WT complex 3D structure and the specification of interaction chains and the mutated residue. Therefore, a separate mutant PDB is not required. In MutaBind2 outputs, positive and negative ΔΔG values correspond to mutations decreasing and increasing binding affinity, respectively, and we considered the cutoff ±1.5 kcal/mol classifying a meaningful effect, which is the server criterion [54].

### 4.7. Search for Potential Diseases Related to the ATP2B Gene Alteration

To assess potential links between the substitutions evaluated at the PMCA-CaM interface that impair pump function and human disease phenotypes, we integrated protein-expression data with gene-disease resources, focusing on Ca^2+^ dysregulation. First, we queried the Human Protein Atlas (HPA) (https://www.proteinatlas.org/ (accessed on 21 March 2025)) [55] for each PMCA isoform using the corresponding gene symbols (*ATP2B1*-*ATP2B4*, which correspond to the PMCA1–4 isoforms, respectively) and recorded tissues and cell types annotated with “High” protein expression in a qualitative manner. Second, we retrieved gene-disease associations (GDAs) for *ATP2B1*-*ATP2B4* from DisGeNET via its interactive API console. We retained associations classified as “GeneticVariation” and “CausalMutation”, and used the DisGeNET ScoreGDA as the primary measure of gene-disease association strength. This 0–1 confidence score integrates the amount and quality of supporting evidence across curated databases and text-mined literature, as well as other sources. Higher values indicate stronger and more reliable associations. We applied thresholds of ≥0.3 for general analyses and ≥0.5 as high-confidence sensitivity. For each disease, we also extracted the total number of genes linked to that disease (NgenesD) in DisGeNET (https://disgenet.com/ (accessed on 9 June 2025)) as a proxy for polygenicity and stratified diseases using an empirical split at ≤30 vs. >30 genes (an operational criterion for visualization and prioritization). To further characterize specificity/pleiotropy, we referenced the DSI/DPI indices as defined in DisGeNET [56].

To relate disease associations to biological context, we assigned each disease to affected organ/system categories by resolving the disease to its MONDO code (Monarch Initiative) (https://monarchinitiative.org/ (accessed on 11 June 2025)) [57] and using available phenotypic annotations (HPO) and ontology hierarchy to map to organ-system classes. When needed, term resolution and cross-links were verified using the EMBL-EBI Ontology Lookup Service (OLS) (https://www.ebi.ac.uk/ols4/ (accessed on 11 June 2025)) [58]. All resources were accessed in June 2025. Results were interpreted as alignment between tissue expression from the HPA and disease systems from the Monarch/OLS, rather than evidence of tissue causality per se.

## 5. Conclusions

In summary, our in silico analysis of six substitutions within the CaMBD across multiple PMCA isoforms reveals it to be a highly sensitive regulatory domain. The predicted effects are dependent on the subregion, residue position, and isoform. Most substitutions classified as radical are expected to weaken CaM binding, with the largest effects concentrated toward the C-terminal portion of the CaMBD. Such perturbations could disrupt Ca^2+^ clearance and signal shaping in cells with high PMCA expression, affecting not only the nervous system but also other tissues. In-depth insight into the effects of these substitutions will require targeted in vitro experiments and comparative analyses of mutations both within the CaMBD and in other regulatory domains of the pump, to delineate their relative contributions to Ca^2+^ dysregulation at the molecular, cellular, and systemic levels.

## Figures and Tables

**Table 1:** Selection of residues for amino acid substitutions.

Isoform/Triplets	T01	T02	T03	T04	T05	T06	T07	T08	T09	T10	T11	T12	T13	T14	T15	T16	T17	T18
PMCA1a [NP_001001323]	tgg (W)	ttt (F)	aga (R)	ggt (G)	ctg (L)	aac (N)	aga (R)	atc (I)	caa (Q)	aca (T)	cag (Q)	atg (M)	gat (D)	gta (V)	gtg (V)	aat (N)	gct (A)	ttc (F)
PMCA1b [NP_001353449.1]	tgg (W)	ttt (F)	aga (R)	ggt (G)	ctg (L)	aac (N)	aga (R)	atc (I)	caa (Q)	aca (T)	cag (Q)	att (I)	cga (R)	gtg (V)	gtg (V)	aat (N)	gca (A)	ttt (F)
PMCA4a [NP_001001396.1]	tgg (W)	ttc (F)	cgg (R)	ggc (G)	ctg (L)	aac (N)	cgt (R)	atc (I)	cag (Q)	act (T)	cag (Q)	atc (I)	gac (D)	gta (V)	att (I)	aac (N)	aca (T)	ttc (F)
PMCA4c [NP_001352712.1]	tgg (W)	ttc (F)	cgg (R)	ggc (G)	ctg (L)	aac (N)	cgt (R)	atc (I)	cag (Q)	act (T)	cag (Q)	atc (I)	gac (D)	gta (V)	att (I)	aac (N)	aca (T)	ttc (F)
PMCA2c [NP_001317540.1]	tgg (W)	ttc (F)	cga (R)	ggc (G)	ctg (L)	aat (N)	cgg (R)	atc (I)	cag (Q)	aca (T)	cag (Q)	att (I)	gaa (E)	gtc (V)	gtc (V)	aat (N)	act (T)	ttc (F)
PMCA2d [NP_001350791.1]	tgg (W)	ttc (F)	cga (R)	ggc (G)	ctg (L)	aat (N)	cgg (R)	atc (I)	cag (Q)	aca (T)	cag (Q)	att (I)	gaa (E)	gta (V)	gtc (V)	aat (N)	act (T)	ttc (F)
PMCA3a [NP_001375289.1]	tgg (W)	ttc (F)	cgg (R)	ggc (G)	ctg (L)	aac (N)	cgg (R)	att (I)	cag (Q)	acg (T)	cag (Q)	atg (M)	gag (E)	gta (V)	gtg (V)	agt (S)	acc (T)	ttc (F)
PMCA4b [NP_001675.3]	tgg (W)	ttc (F)	cgg (R)	ggc (G)	ctg (L)	aac (N)	cgt (R)	atc (I)	cag (Q)	act (T)	cag (Q)	atc (I)	aaa (K)	gtg (V)	gtc (V)	aaa (K)	gcg (A)	ttc (F)
PMCA2a [NP_001001331.1]	tgg (W)	ttc (F)	cga (R)	ggc (G)	ctg (L)	aat (N)	cgg (R)	atc (I)	cag (Q)	aca (T)	cag (Q)	atc (I)	cgc (R)	gtc (V)	gtg (V)	aag (K)	gcg (A)	ttc (F)
PMCA2b [NP_001340493.1]	tgg (W)	ttc (F)	cga (R)	ggc (G)	ctg (L)	aat (N)	cgg (R)	atc (I)	cag (Q)	aca (T)	cag (Q)	atc (I)	cgc (R)	gtc (V)	gtg (V)	aag (K)	gcg (A)	ttc (F)
PMCA3b [NP_001001344.1]	tgg (W)	ttc (F)	cgg (R)	ggc (G)	ctg (L)	aac (N)	cgg (R)	att (I)	cag (Q)	acg (T)	cag (Q)	atc (I)	cgg (R)	gtg (V)	gtg (V)	aaa (K)	gcg (A)	ttc (F)
Nucleotide conservation	***	**	*	**	***	**	*	**	**	**	***	**		**	*	*	*	**
Original Residue					L	N		I						V				F
Potential changes in the second position of the triplet					cgg	atc/att		acc/act						gaa/gag/gac				tct/tcc
Final residue after nucleotide changes					R	I		T						E/D				S

Proposed edits list the original codon, the single nucleotide substitution (position indicated in red), and the resulting amino acid change. Nucleotide substitutions target the second base of the codon. Asterisks (*, ** and ***) indicate the nucleotide conservation in each position. T: codon for each amino acid residue.

**Table 2:** Confidence scores of CaMBD-PMCA predicted complexes using AlphaFold3.

Wild-Type Models						
**Isoform WT Group**	**ipTM**					
	**Model 0**	**Model 1**	**Model 2**	**Model 3**	**Model 4**	**IC_95_**
PMCA1a	0.71	0.68	0.67	0.65	0.65	[0.64–0.7]
PMCA1b	0.71	0.73	0.68	0.66	0.61	[0.62–0.74]
PMCA2a/2b/3b	0.73	0.67	0.69	0.63	0.62	[0.61–0.72]
PMCA2/c/2d	0.59	0.53	0.54	0.55	0.53	[0.52–0.58]
PMCA3a	0.64	0.62	0.58	0.55	0.55	[0.54–0.64]
PMCA4a/4c	0.64	0.61	0.59	0.59	0.56	[0.56–0.63]
PMCA4b	0.74	0.65	0.65	0.62	0.59	[0.58–0.72]
**Literature-Reported Mutations**						
	**Model 0**	**Model 1**	**Model 2**	**Model 3**	**Model 4**	**IC_95_**
PMCA2b V → F	0.69	0.71	0.70	0.60	0.62	[0.6–0.73]
PMCA3b W → A	0.66	0.63	0.59	0.61	0.55	[0.56–0.66]
PMCA3b G → D	0.71	0.69	0.71	0.71	0.68	[0.68–0.72]
**Evaluated Substitutions**						
**L5R**	**Model 0**	**Model 1**	**Model 2**	**Model 3**	**Model 4**	**IC_95_**
PMCA1a	0.69	0.67	0.66	0.67	0.62	[0.63–0.69]
PMCA1b	0.73	0.66	0.68	0.66	0.61	[0.61–0.72]
PMCA2a/2b/3b	0.8	0.7	0.68	0.68	0.68	[0.64–0.77]
PMCA2/c/2d	0.62	0.59	0.6	0.55	0.57	[0.55–0.62]
PMCA3a	0.68	0.55	0.61	0.56	0.58	[0.53–0.66]
PMCA4a/4c	0.67	0.62	0.63	0.6	0.41	[0.46–0.71]
PMCA4b	0.67	0.64	0.65	0.64	0.62	[0.62–0.67]
**N6I**	**Model 0**	**Model 1**	**Model 2**	**Model 3**	**Model 4**	**IC_95_**
PMCA1a	0.71	0.71	0.63	0.63	0.6	[0.59–0.72]
PMCA1b	0.68	0.65	0.63	0.65	0.61	[0.61–0.68]
PMCA2a/2b/3b	0.68	0.62	0.61	0.57	0.58	[0.56–0.67]
PMCA2/c/2d	0.65	0.62	0.57	0.57	0.4	[0.44–0.68]
PMCA3a	0.65	0.6	0.63	0.61	0.35	[0.41–0.72]
PMCA4a/4c	0.57	0.61	0.55	0.58	0.53	[0.53–0.61]
PMCA4b	0.7	0.59	0.56	0.56	0.54	[0.51–0.67]
**I8T**	**Model 0**	**Model 1**	**Model 2**	**Model 3**	**Model 4**	**IC_95_**
PMCA1a	0.82	0.73	0.73	0.68	0.62	[0.62–0.81]
PMCA1b	0.79	0.72	0.72	0.7	0.74	[0.69–0.78]
PMCA2a/2b/3b	0.75	0.74	0.72	0.68	0.43	[0.5–0.83]
PMCA2/c/2d	0.63	0.63	0.63	0.63	0.58	[0.59–0.65]
PMCA3a	0.67	0.66	0.65	0.65	0.64	[0.64–0.67]
PMCA4a/4c	0.69	0.67	0.62	0.62	0.6	[0.59–0.69]
PMCA4b	0.7	0.63	0.61	0.58	0.62	[0.57–0.68]
**V14E/D**	**Model 0**	**Model 1**	**Model 2**	**Model 3**	**Model 4**	**IC_95_**
PMCA1a	0.6	0.59	0.6	0.58	0.54	[0.55–0.61]
PMCA1b	0.62	0.54	0.54	0.56	0.4	[0.43–0.63]
PMCA2a/2b/3b	0.61	0.59	0.57	0.55	0.56	[0.55–0.61]
PMCA2/c/2d	0.56	0.56	0.52	0.39	0.38	[0.37–0.59]
PMCA3a	0.59	0.56	0.54	0.55	0.52	[0.52–0.58]
PMCA4a/4c	0.53	0.53	0.42	0.35	0.37	[0.33–0.55]
PMCA4b	0.54	0.53	0.51	0.49	0.41	[0.43–0.56]
**F18S**	**Model 0**	**Model 1**	**Model 2**	**Model 3**	**Model 4**	**IC_95_**
PMCA1a	0.69	0.69	0.64	0.62	0.59	[0.59–0.7]
PMCA1b	0.71	0.72	0.69	0.67	0.61	[0.63–0.73]
PMCA2a/2b/3b	0.64	0.64	0.6	0.43	0.42	[0.41–0.68]
PMCA2/c/2d	0.68	0.6	0.59	0.63	0.6	[0.57–0.67]
PMCA3a	0.65	0.61	0.6	0.59	0.58	[0.57–0.64]
PMCA4a/4c	0.66	0.61	0.61	0.6	0.4	[0.45–0.7]
PMCA4b	0.58	0.6	0.57	0.47	0.45	[0.45–0.62]

**Table 3:** Energy binding variation for each PMCA CaMBD-CaM complex.

	Evaluated Substitutions										Literature Reported Mutations		
	FoldX					MutaBind2							
	Pattern 1			Pattern 2		Pattern 1			Pattern 2				
	L5R	N6I	I8T	V14E/D	F18S	L5R	N6I	I8T	V14E/D	F18S			
PMCA (Isoform)/Gibbs Function	ΔΔG	ΔΔG	ΔΔG	ΔΔG	ΔΔG	ΔΔG	ΔΔG	ΔΔG	ΔΔG	ΔΔG	PMCA (Isoform)	FoldX ΔΔG	MutaBind2 ΔΔG
PMCA1a	−3.92	−0.72	−4.14	3.42	1.17	1.98	0.32	0.32	2.95	5.04	PMCA2b (V → F)	1.98	4.24
PMCA1b	−0.69	−4.97	5.82	3.15	5.64	0.21	0.31	2.5	3.13	4.23	PMCA3b (W → A)	15.88	3.51
PMCA2a_2b_3b	8.01	2.91	7.09	7.29	6.59	1.9	0.85	2.53	3.67	4.2	PMCA3b (G → D)	9.29	2.39
PMCA2c_2d	0.22	−8.28	2.21	9.19	4.99	1.72	0.72	2.57	3.77	4.33			
PMCA3a	3.11	1.15	4.59	4.99	0.19	2.6	1.56	2.9	3.35	4.57			
PMCA4a_4c	−2.79	1.72	3.77	12.05	7.9	1.85	0.05	2.97	3.45	4.59			
PMCA4b	2.6	8.06	5.43	16.12	3.73	1.92	0.22	2.77	2.91	4.61			

In the case of FoldX values, the calculations were based on the Alphafold Model 0. Values expressed in kcal/mol.

## Data Availability

The original contributions presented in this study are included in the article/Supplementary Materials. Further inquiries can be directed to the corresponding author.

## Supplementary Materials

The following supporting information can be downloaded at: https://www.mdpi.com/article/10.3390/ijms262311750/s1.

## Abbreviations

The following abbreviations are used in this manuscript:

PMCA	Plasma-membrane Ca^2+^-ATPase
CaMBD	Calmodulin Binding Domain
CaM	Calmodulin
ipTM	Inter-residue predicted TM-score
WT	Wild-type
NMR	Nuclear magnetic resonance
RMSD	Root mean square deviation
pLDDT	Predicted local distance difference test
RefSeq	NCBI Reference Sequences
ΔG	Change in Gibbs Energy
GDA	Gene-disease associations
NgenesD	Total number of genes linked to that disease
DSI/DPI	Specificity/pleiotropy
HPO	Phenotypic annotations
OLS	Ontology Lookup Service
HPA	Human Protein Atlas
ACMG-AMP	American College of Medical Genetics and Genomics-Association for Molecular Pathology
