# Supplementary material for: Calmodulin Interaction Interface with Plasma Membrane Ca2+-ATPase Isoforms: An Integrative Bioinformatic Analysis
Source: Int J Mol Sci. 2025 Dec 4;26(23):11750. doi: 10.3390/ijms262311750 (PMC12693652; doi:10.3390/ijms262311750)
Supplement: Supplementary file 1 [file ijms-26-11750-s001.zip › Supplementary Materials Files S3.pptx]

## Slide 1
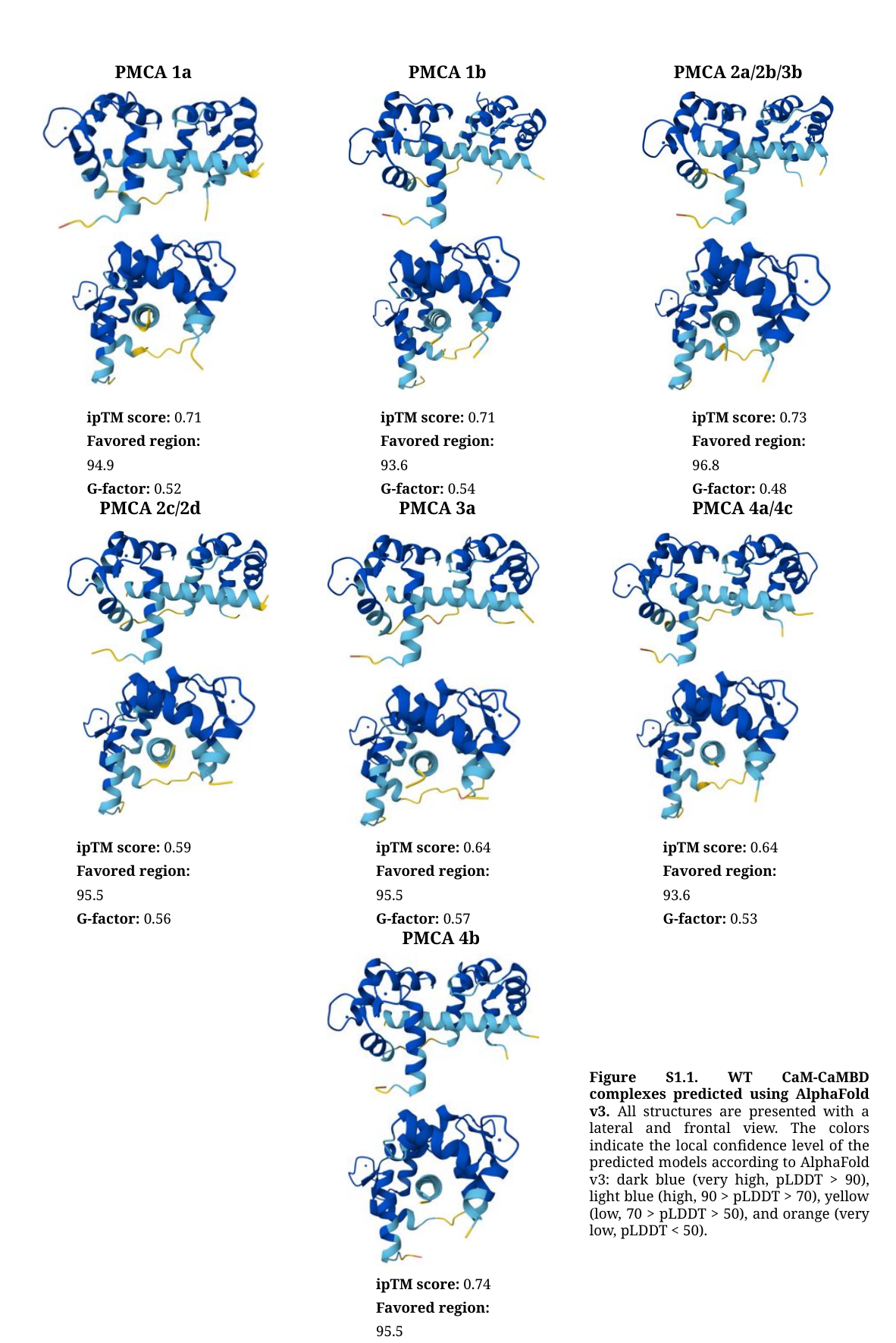

PMCA 1a
PMCA 1b
PMCA 2a/2b/3b
ipTM score: 0.71
Favored region: 94.9
G-factor: 0.52
ipTM score: 0.71
Favored region: 93.6
G-factor: 0.54
ipTM score: 0.73
Favored region: 96.8
G-factor: 0.48
PMCA 2c/2d
PMCA 3a
PMCA 4a/4c
ipTM score: 0.59
Favored region: 95.5
G-factor: 0.56
ipTM score: 0.64
Favored region: 95.5
G-factor: 0.57
ipTM score: 0.64
Favored region: 93.6
G-factor: 0.53
PMCA 4b
Figure S1.1. WT CaM-CaMBD complexes predicted using AlphaFold v3. All structures are presented with a lateral and frontal view. The colors indicate the local confidence level of the predicted models according to AlphaFold v3: dark blue (very high, pLDDT > 90), light blue (high, 90 > pLDDT > 70), yellow (low, 70 > pLDDT > 50), and orange (very low, pLDDT < 50).
ipTM score: 0.74
Favored region: 95.5
G-factor: 0.55

## Slide 2
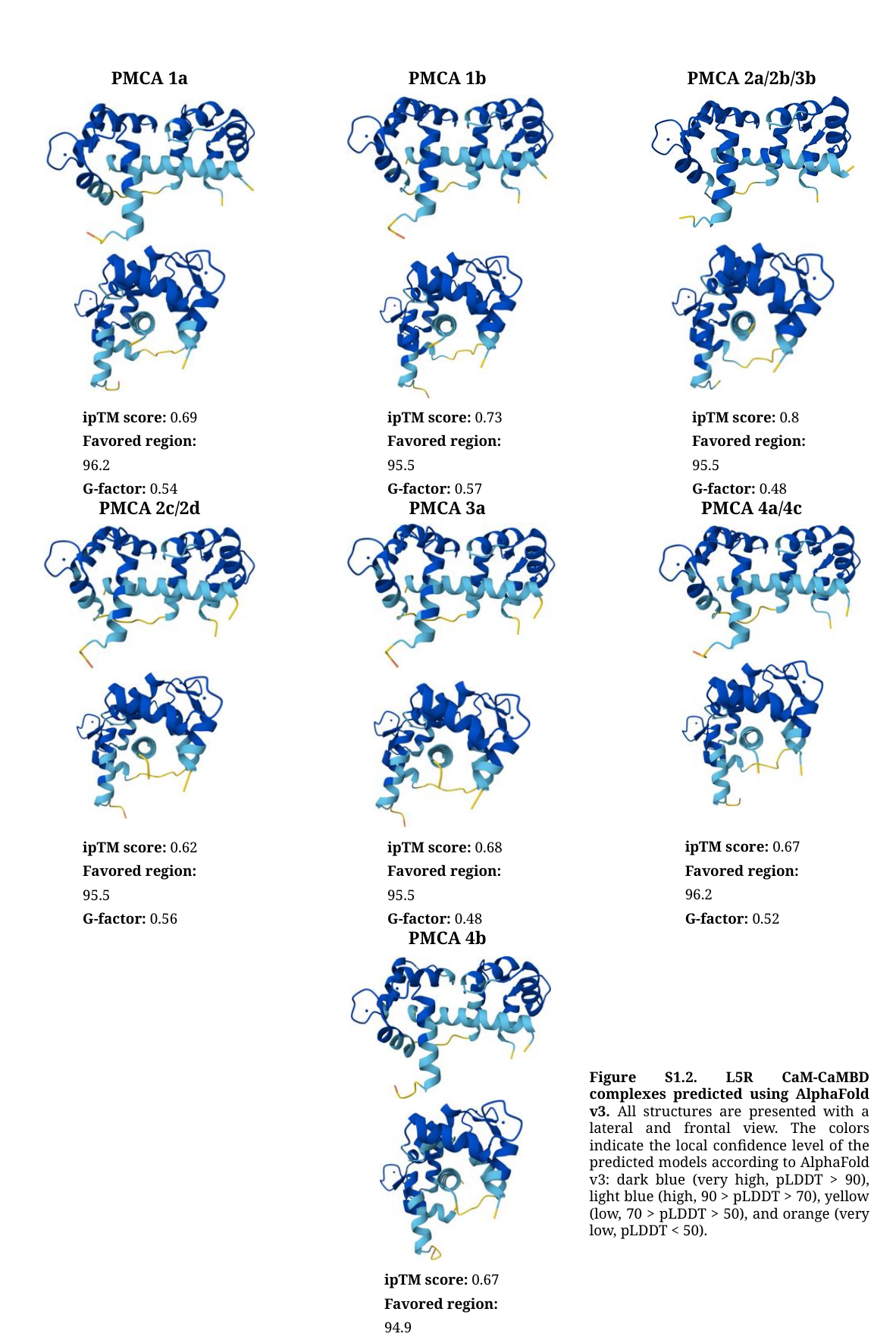

PMCA 1a
PMCA 1b
PMCA 2a/2b/3b
ipTM score: 0.69
Favored region: 96.2
G-factor: 0.54
ipTM score: 0.73
Favored region: 95.5
G-factor: 0.57
ipTM score: 0.8
Favored region: 95.5
G-factor: 0.48
PMCA 2c/2d
PMCA 3a
PMCA 4a/4c
ipTM score: 0.67
Favored region: 96.2
G-factor: 0.52
ipTM score: 0.62
Favored region: 95.5
G-factor: 0.56
ipTM score: 0.68
Favored region: 95.5
G-factor: 0.48
PMCA 4b
Figure S1.2. L5R CaM-CaMBD complexes predicted using AlphaFold v3. All structures are presented with a lateral and frontal view. The colors indicate the local confidence level of the predicted models according to AlphaFold v3: dark blue (very high, pLDDT > 90), light blue (high, 90 > pLDDT > 70), yellow (low, 70 > pLDDT > 50), and orange (very low, pLDDT < 50).
ipTM score: 0.67
Favored region: 94.9
G-factor: 0.56

## Slide 3
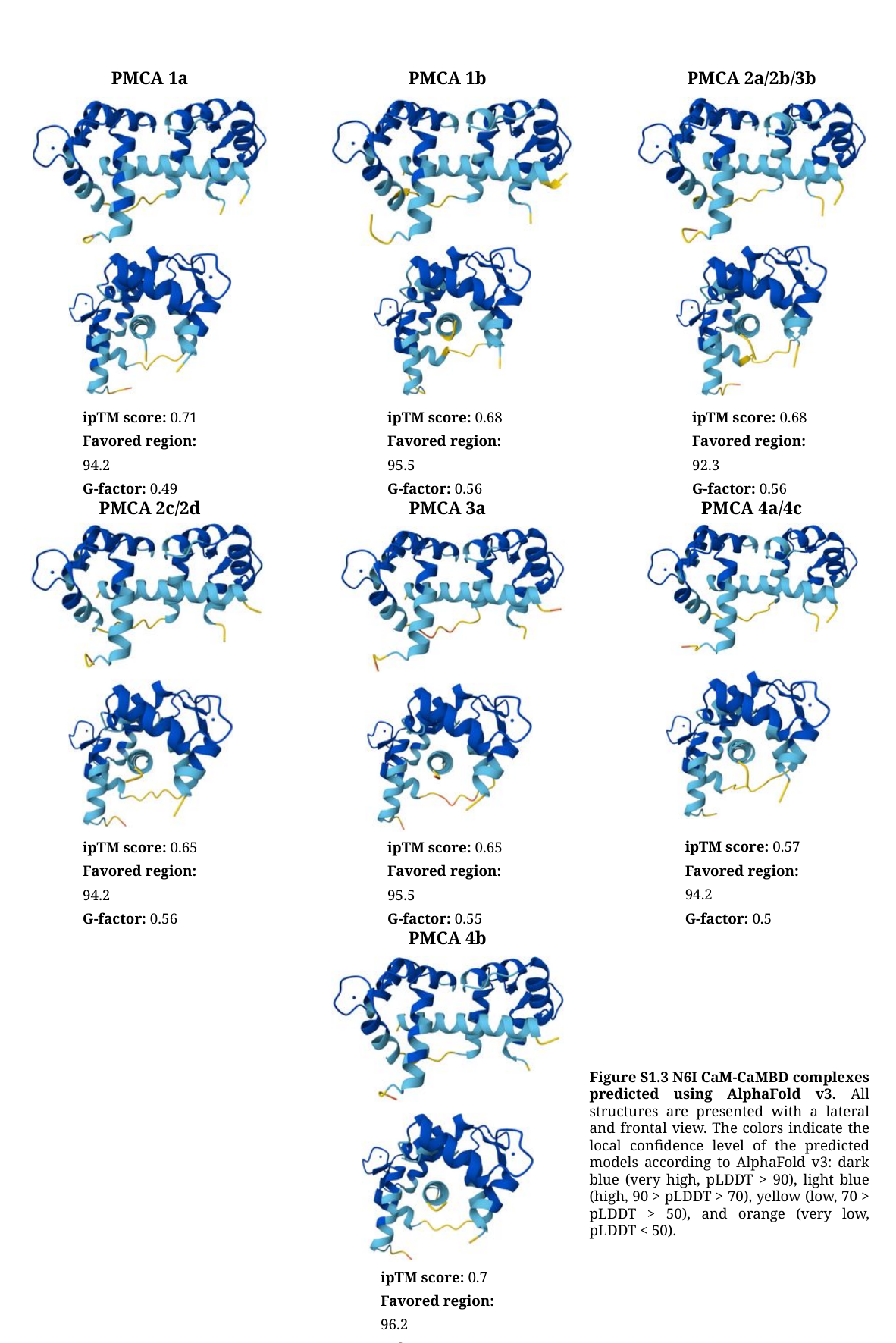

PMCA 1a
PMCA 1b
PMCA 2a/2b/3b
ipTM score: 0.71
Favored region: 94.2
G-factor: 0.49
ipTM score: 0.68
Favored region: 95.5
G-factor: 0.56
ipTM score: 0.68
Favored region: 92.3
G-factor: 0.56
PMCA 2c/2d
PMCA 3a
PMCA 4a/4c
ipTM score: 0.57
Favored region: 94.2
G-factor: 0.5
ipTM score: 0.65
Favored region: 94.2
G-factor: 0.56
ipTM score: 0.65
Favored region: 95.5
G-factor: 0.55
PMCA 4b
Figure S1.3 N6I CaM-CaMBD complexes predicted using AlphaFold v3. All structures are presented with a lateral and frontal view. The colors indicate the local confidence level of the predicted models according to AlphaFold v3: dark blue (very high, pLDDT > 90), light blue (high, 90 > pLDDT > 70), yellow (low, 70 > pLDDT > 50), and orange (very low, pLDDT < 50).
ipTM score: 0.7
Favored region: 96.2
G-factor: 0.56

## Slide 4
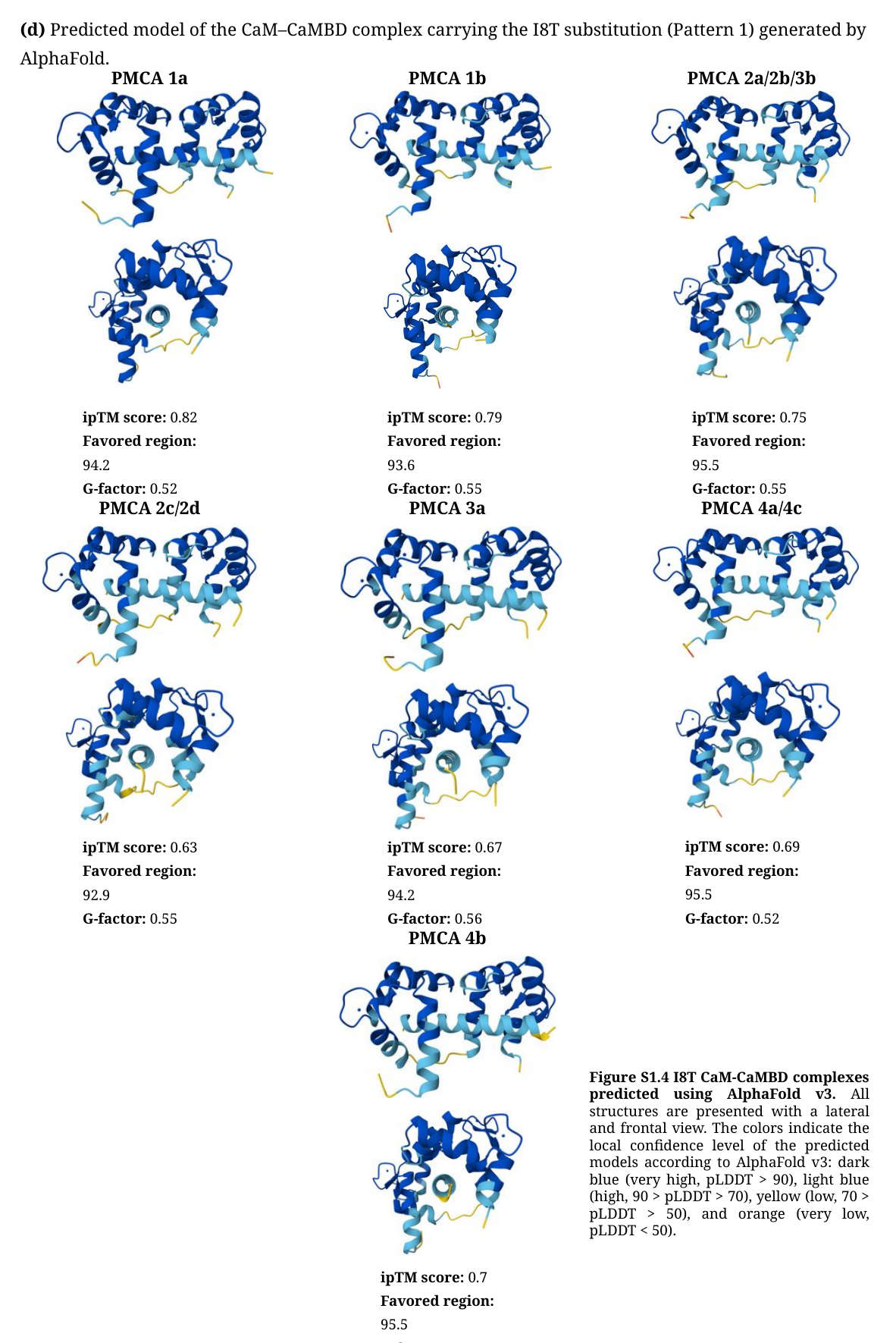

(d) Predicted model of the CaM–CaMBD complex carrying the I8T substitution (Pattern 1) generated by AlphaFold.
PMCA 1a
PMCA 1b
PMCA 2a/2b/3b
ipTM score: 0.82
Favored region: 94.2
G-factor: 0.52
ipTM score: 0.79
Favored region: 93.6
G-factor: 0.55
ipTM score: 0.75
Favored region: 95.5
G-factor: 0.55
PMCA 2c/2d
PMCA 3a
PMCA 4a/4c
ipTM score: 0.69
Favored region: 95.5
G-factor: 0.52
ipTM score: 0.63
Favored region: 92.9
G-factor: 0.55
ipTM score: 0.67
Favored region: 94.2
G-factor: 0.56
PMCA 4b
Figure S1.4 I8T CaM-CaMBD complexes predicted using AlphaFold v3. All structures are presented with a lateral and frontal view. The colors indicate the local confidence level of the predicted models according to AlphaFold v3: dark blue (very high, pLDDT > 90), light blue (high, 90 > pLDDT > 70), yellow (low, 70 > pLDDT > 50), and orange (very low, pLDDT < 50).
ipTM score: 0.7
Favored region: 95.5
G-factor: 0.55

## Slide 5
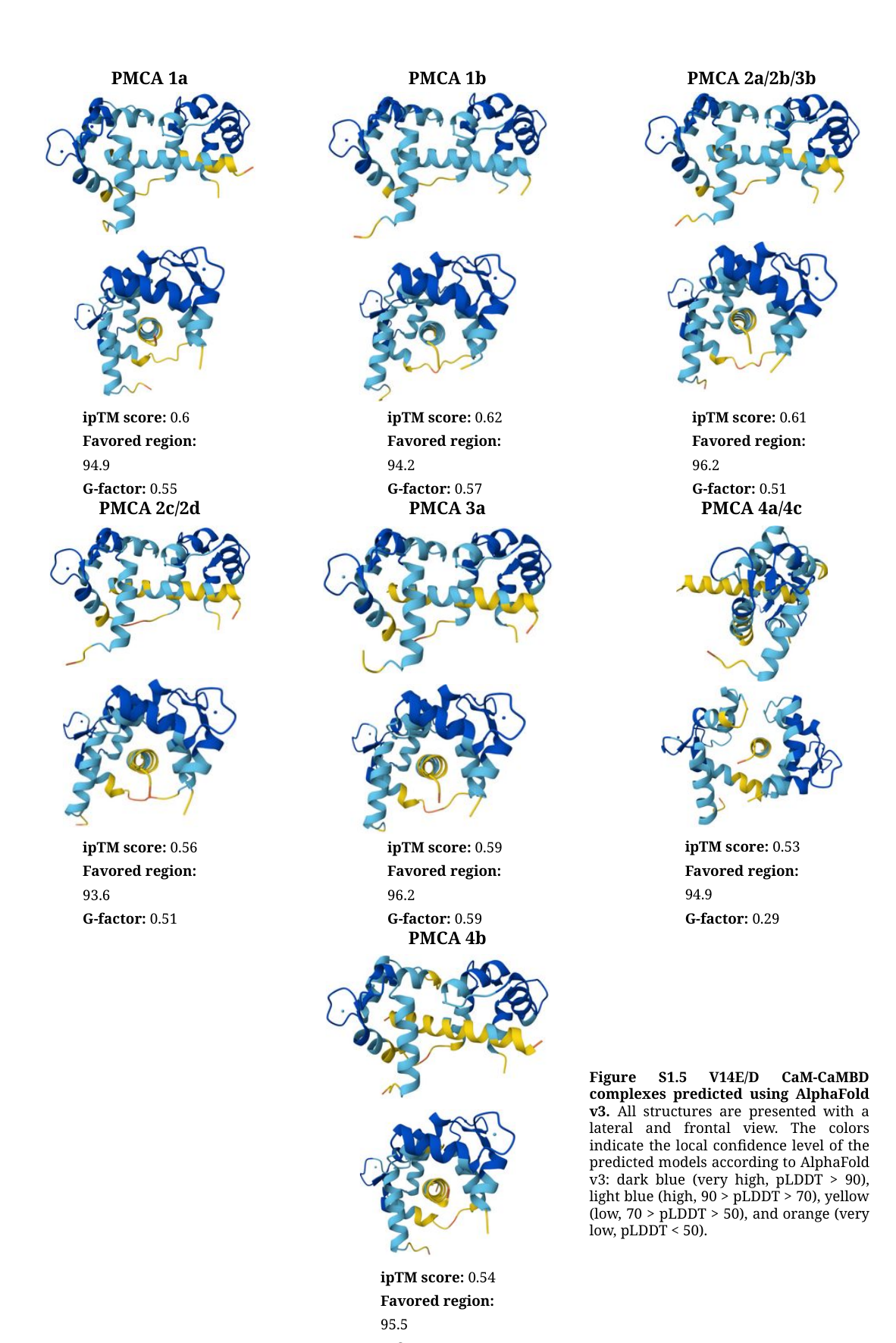

PMCA 1a
PMCA 1b
PMCA 2a/2b/3b
ipTM score: 0.6
Favored region: 94.9
G-factor: 0.55
ipTM score: 0.62
Favored region: 94.2
G-factor: 0.57
ipTM score: 0.61
Favored region: 96.2
G-factor: 0.51
PMCA 2c/2d
PMCA 3a
PMCA 4a/4c
ipTM score: 0.53
Favored region: 94.9
G-factor: 0.29
ipTM score: 0.56
Favored region: 93.6
G-factor: 0.51
ipTM score: 0.59
Favored region: 96.2
G-factor: 0.59
PMCA 4b
Figure S1.5 V14E/D CaM-CaMBD complexes predicted using AlphaFold v3. All structures are presented with a lateral and frontal view. The colors indicate the local confidence level of the predicted models according to AlphaFold v3: dark blue (very high, pLDDT > 90), light blue (high, 90 > pLDDT > 70), yellow (low, 70 > pLDDT > 50), and orange (very low, pLDDT < 50).
ipTM score: 0.54
Favored region: 95.5
G-factor: 0.55

## Slide 6
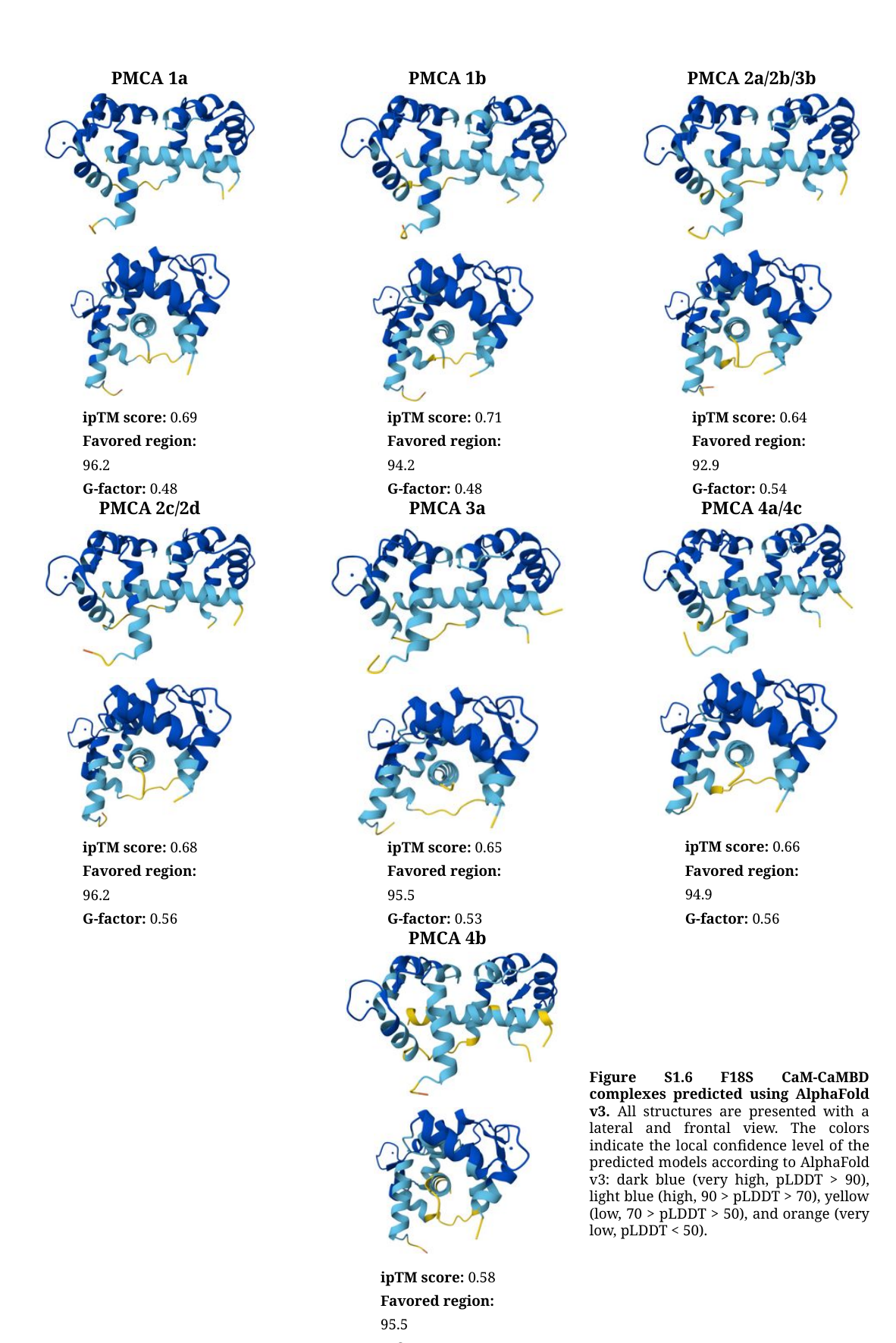

PMCA 1a
PMCA 1b
PMCA 2a/2b/3b
ipTM score: 0.69
Favored region: 96.2
G-factor: 0.48
ipTM score: 0.71
Favored region: 94.2
G-factor: 0.48
ipTM score: 0.64
Favored region: 92.9
G-factor: 0.54
PMCA 2c/2d
PMCA 3a
PMCA 4a/4c
ipTM score: 0.66
Favored region: 94.9
G-factor: 0.56
ipTM score: 0.68
Favored region: 96.2
G-factor: 0.56
ipTM score: 0.65
Favored region: 95.5
G-factor: 0.53
PMCA 4b
Figure S1.6 F18S CaM-CaMBD complexes predicted using AlphaFold v3. All structures are presented with a lateral and frontal view. The colors indicate the local confidence level of the predicted models according to AlphaFold v3: dark blue (very high, pLDDT > 90), light blue (high, 90 > pLDDT > 70), yellow (low, 70 > pLDDT > 50), and orange (very low, pLDDT < 50).
ipTM score: 0.58
Favored region: 95.5
G-factor: 0.51
