# Supplementary material for: Calmodulin Interaction Interface with Plasma Membrane Ca2+-ATPase Isoforms: An Integrative Bioinformatic Analysis
Source: Int J Mol Sci. 2025 Dec 4;26(23):11750. doi: 10.3390/ijms262311750 (PMC12693652; doi:10.3390/ijms262311750)
Supplement: Supplementary file 1 [file ijms-26-11750-s001.zip › Supplementary Materials Files S4.pptx]

## Slide 1
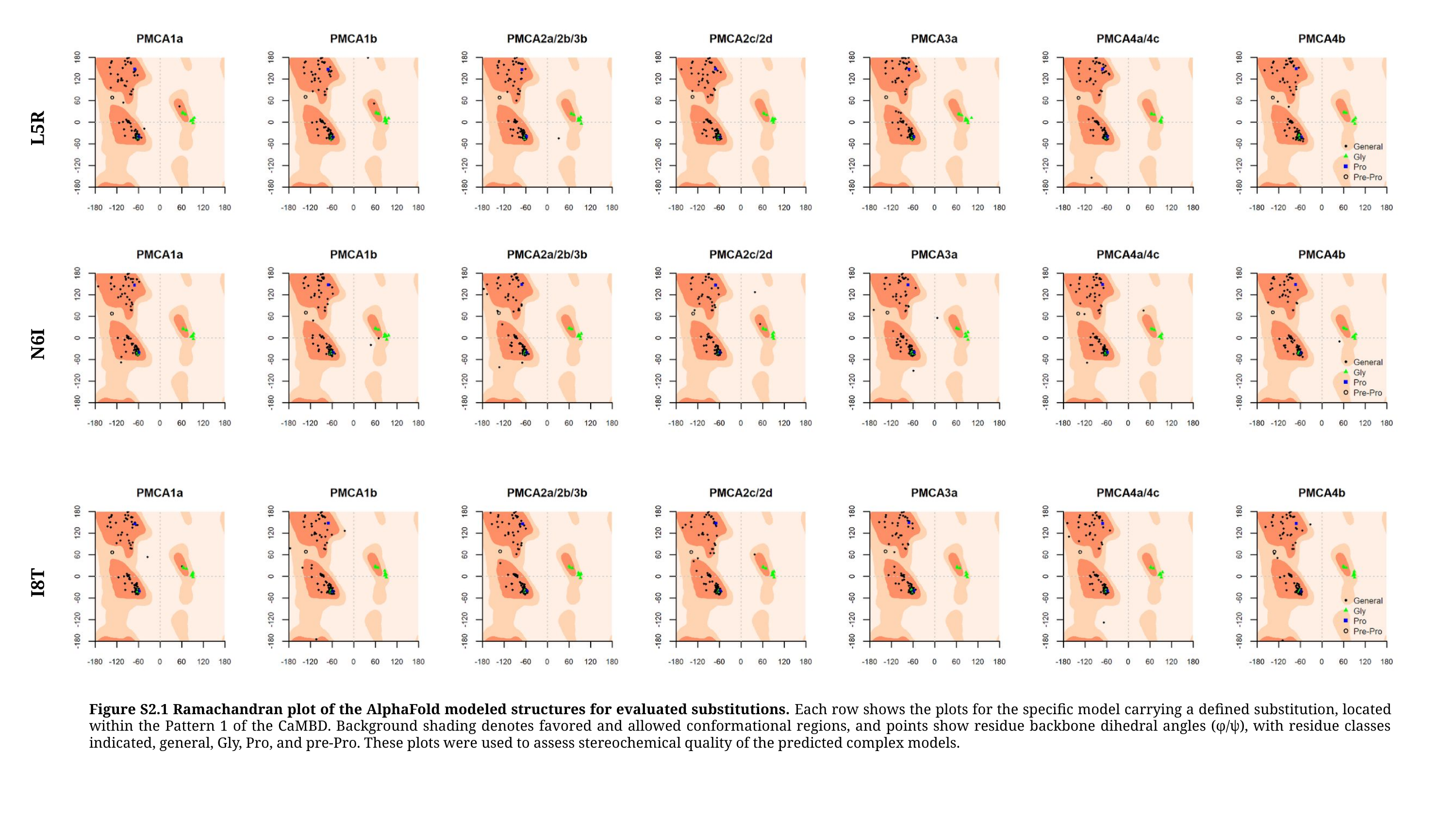

L5R
N6I
I8T
Figure S2.1 Ramachandran plot of the AlphaFold modeled structures for evaluated substitutions. Each row shows the plots for the specific model carrying a defined substitution, located within the Pattern 1 of the CaMBD. Background shading denotes favored and allowed conformational regions, and points show residue backbone dihedral angles (φ/ψ), with residue classes indicated, general, Gly, Pro, and pre-Pro. These plots were used to assess stereochemical quality of the predicted complex models.

## Slide 2
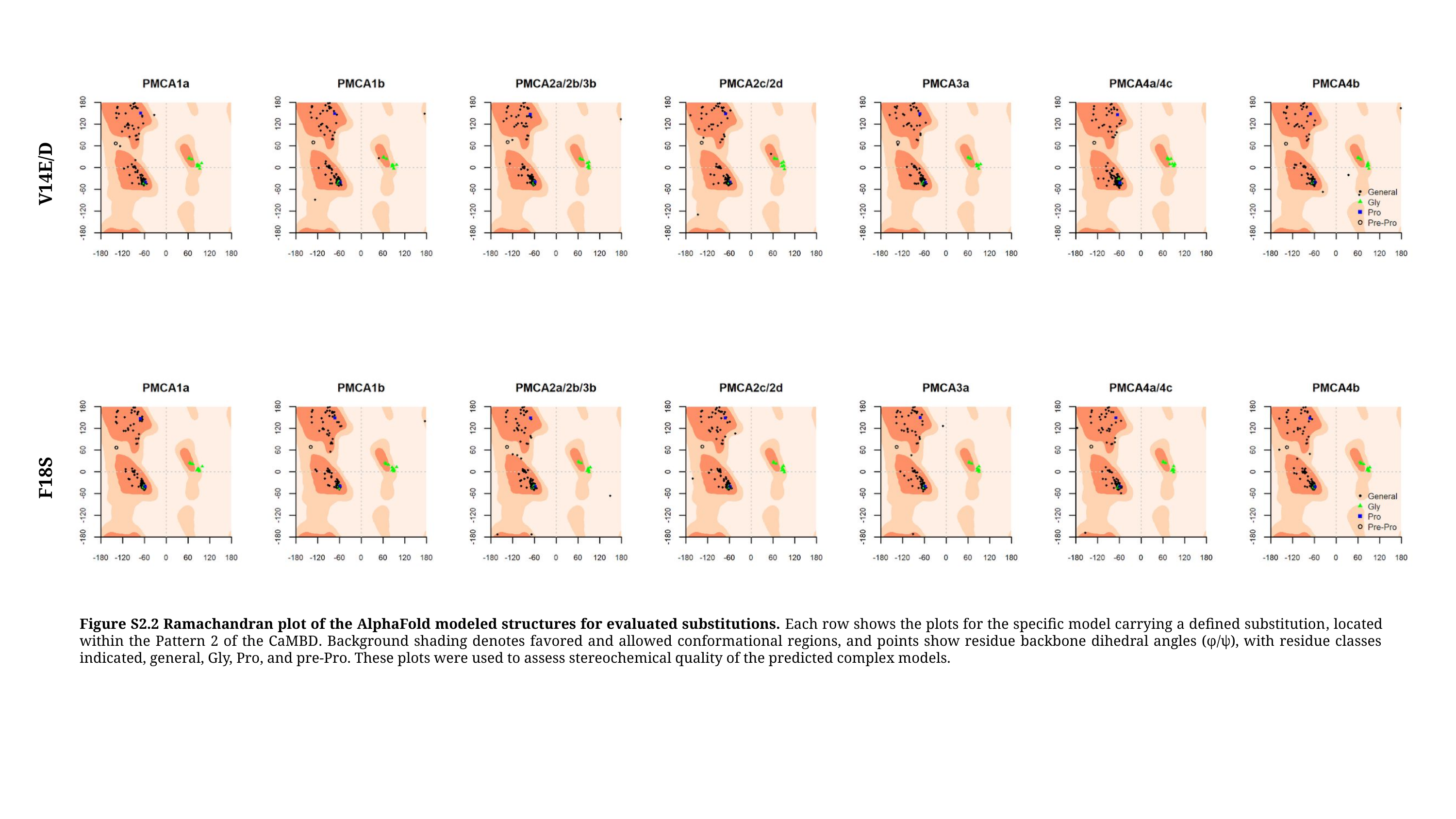

V14E/D
F18S
Figure S2.2 Ramachandran plot of the AlphaFold modeled structures for evaluated substitutions. Each row shows the plots for the specific model carrying a defined substitution, located within the Pattern 2 of the CaMBD. Background shading denotes favored and allowed conformational regions, and points show residue backbone dihedral angles (φ/ψ), with residue classes indicated, general, Gly, Pro, and pre-Pro. These plots were used to assess stereochemical quality of the predicted complex models.
